# Washington's Teeth

**Published:** 1876-07

**Authors:** 


### ARTICLE VI.

### *Washington's Teeth.*

The Father of his Country had only one false thing about him. That was a set of false teeth. He wore a double set. I have them in one hand while writing you these lines with the other.

That Washington wore false teeth, and that they were very roughly made, will explain some things in regard to the expression of his countenance, which has been the subject of discussion. There is a marked difference in the mouth represented in the Peale portrait, and in the Stuart portrait, which has given the most general impression to the world of the style of face. In Stuart's portrait, and in all the engravings made from it and repeated by millions, there is a projecting upper lip, giving an expression of *pouting* which Washington himself did not like, because it was not natural to him.

Gen. G. B. McClellan has the original of a medallion head of Washington which the illustrious man preferred so decidedly that he presented it to Mrs. Morris, with the following words in a note:

"The President's compliments accompany the enclosed to Mrs. Morris."

This portrait has been elegantly engraved under the direction of the late Secretary of War. It shows no projection of the upper lip; the mouth is firm and symmetrical, and the appearance of fulness, as in the other, is wanting.

These differences in the expression are easily explained by the fact that Washington lost all his teeth; had a set of artificial teeth made; they were clumsy and irksome, so that it is quite likely they were sometimes worn and often not: that the position of the lips was taken as they appeared at various times; and the whole expression varies with that of the mouth, which is more of an index of the mind than any other portion of the human countenance. Try the experiment, and see for yourself what a difference it makes as you compress or extend the lips. Lavater will give you all the types of man as indicated by the mouth. Other physiognomists have held that there is a direct relation of the muscles of the lips to the upper lobes of the brain, so that, unless repressed or perverted by artificial means, the mouth will naturally intimate some of the most important mental exercises or peculiarities. A man with false teeth, so set as to change the natural shape of his lips, and to prevent their ready play with the varying moods of the mind, will not be fairly handed down to posterity in a picture that gives him a protuberant mouth, unless nature gave him one with his natural teeth.

The history of these false teeth of Washington is not involved in obscurity. And as they are the most nearly personal of anything relating to Washington that survives the grave, they are objects of real interest, and not merely of curiosity. In the Milan Cathedral they preserve with great care, teeth from the head of Abraham, the Father of Faithful; of Daniel, who stopped the mouths of lions; of John also, and the prophet Elisha; and all these are claimed to be the veritable molars of the men whose bones were buried long ages agone.

But it is not pretended that these are the teeth that Washington had in his head when he had the hatchet in his hand. By the way, if you have any doubt as to the truth of the hatchet story in the early life of G. W., let me just say that I have a piece of the cherry tree which he hacked; of course, the hacking required a hatchet, and thus the historical incident, so valuable in juvenile instruction, is put beyond reasonable dispute. With unreasonable people there is no use of reasoning.

At what period in his eventful life, our great Washington first lost his old teeth and got these new ones, does not appear in any biographies in my possession. But it was not until the year before his death that Washington addressed to Mr. Greenwood, a dentist of New York, the following letter, in which the defects of the work are set forth and improvements suggested with that accurate detail and perspicuity which distinguish the writings of this wonderful man:

PHILADELPHIA, 12th Dec., 1798.

SIR: Your letter of the 8th came safe, and as I am hurrying in order to leave this city to-morrow, I must be short The principal thing you will have to attend to in the alteration you are about to make, is to let the upper bar fall back from the lower one ; whether the teeth are quite straight, or inclining a little in, or a little rounding outwards, this is immaterial ; for I find it is the bars alone, both above and below, that give the lips the pouting and swelling appearance—of consequence, if this can be remedied, all will be well.

I send you the old bars, which you returned to me with the new set, because you have desired. But they may be destroyed, or anything else done with them you please, for you will find that I have been obliged to file them away so much above, to remedy the evil I have been complaining of, as to render them useless, perhaps, to receive new teeth. But of this you are better able to judge than I am. If you can fix the teeth (not only the new bars which you have) on the old bars which you will receive with this letter, I should prefer it, because the latter are easy in the mouth—and you will perceive, moreover, that when the edges of the upper and lower teeth are put together, that the upper falls back into the mouth, which they ought to do, or it will have the effect of forcing the lip out just under the nose.

I shall only repeat again that I feel much obliged by your extreme willingness and readiness to accommodate me, and that I am, Sir, Your obed't servant

GEO. WASHINGTON.

MR. JNO. GREENWOOD.

"The letter was written to John Greenwood, of New York, who had a younger brother, William Pitt Greenwood, in Boston, both skilled in the practice of dentistry as then understood.

"The 'bar' referred to took the place of what is now the plate, and was made of ivory carved to an approximate adaptation to the gum, and the teeth were fastened by rivets to the bar. At that time neither the principle of complete support by atmospheric pressure nor by springs was well understood, and the support of an artificial set in the mouth depended somewhat on training of the facial muscles.

"The bulk on the thickness of the bar, together with the action of the muscles in keeping it in place, was apt to give a puff or 'pouting' expression of the face, or some part of it. This, in the case under consideration, was principally evident about the upper lip, as seen in Stuart's portrait of Washington, painted about 1796, while the subject had the set of teeth, the bar of which he had, being at a distance from the dentist, modified so much with his own hands, to obtain comfort, and if possible to correct the objectionable expression mentioned.

"The suggestion in regard to the 'falling back' of the upper teeth, though probably not anatomically correct, even at his age, was evidently made with reference to the same point, as an additional aid in diminishing the pouting expression of the upper lip, and in producing the original symmetry of the face which we find in Houdon's bust, made in 1785, and which is also seen in Peale's portraits.

JACOB L. WILLIAMS."

Observe that Washington specifies the pouting expression as the result of these badly fitting teeth. And to look at them, the wonder is that any man ever held them in his mouth five minutes. The teeth are bits of bone, scarcely trying to look like teeth, attached to gold plate, with strips riveted across to strengthen the teeth in place; while coiled wire at the end of the jaws makes a spring, and assists in opening and closing the machine. They are as near to the perfect work of our day in this line, as the old windmill telegraph to a Morse instrument. The Baltimore Dental College has obtained possession of them, and Drs. J. Allen & Son, of this city, preparing a case of their workmanship for exhibition at the Centennial in Philadelphia, have secured the loan of them to put into the foreground of their magnificent display, that the present generation may see what the Father of his Country endured in the times that tried men's souls. He must have been a man of exemplary patience and fortitude, to have borne with these things in his mouth. As to eating with them, or talking with them in, it would seem impossible, but it is probable they answered the purpose very well, for it is not always the handsomest looking machine that does the best work.—*N. Y. Observer.*

